# Clinical Lectures on Some Disorders Dependent upon Genital Irritation; Proper Treatment of Talipes, Etc.

**Published:** 1880-07

**Authors:** Lewis A. Sayre


### Selections.

---

### CLINICAL LECTURES ON SOME DISORDERS DEPENDENT UPON GENITAL IRRITATION; PROPER TREATMENT OF TALIPES, ETC.

BY LEWIS A. SAYRE, M. D.

*Case I.*—The first case I have to show you to day is one of loss of co-ordination and spastic contraction of the lower extremities, with strongly marked talipes equino-varus in both feet.

The mother gives the following history: The patient's name is Anna, and she is ten years of age. She seemed to be normally developed at birth, though the mother thinks the lower extremities were not proportionately well developed with the trunk. Three months after birth an abscess began to develop in the neck. It was fina ly opened and emptied of its contents. It continued to discharge pus for a long time. She also had some difficulty of vision, but the cause is not very clear. Her health became very delicate and remained so for several years. When she was about thirteen months of age she made some efforts at creeping, and at two and a half years she began to walk some, which was, however, very imperfectly and awkwardly done. As she continued to make efforts at walking her knees began to give way and she became quite knock-kneed. She also no longer put the whole plantar surfaces on the floor, but gradually began to walk "on her toes." In this awkward condition she continued to get around in the room by the aid of assistants, chairs, and beds, until five years of age, when she met with a serious accident She fell a distance of thirty feet, through a skylight. Although no apparent serious damage had been sustained, the child made no more efforts at locomotion for nearly two years. Many efforts have been made by surgeons and at some of the orthopædic institutions of this city to correct her deformities, but with no success worth speaking about.

She is able, by the aid of assistants, to move small distances, or stand up while holding to a chair, and when she does so, she stands on "tip-toe," and her knees are tightly pressed together. The mother states that she has been annoyed, constantly from her birth to the present time by thread worms (oxyuris vermicularis), making her existence almost unbearable. They accumulate in the rectum in great numbers. They torture her so that the mother has to use enemas, or extract them with her finger. They not only produce great irritation in the rectum, but voluntarily leave that abode and get on the verge of the anus and into the genital tract. The little girl has always suffered from genital irritation. She desires to urinate often, and suffers considerable pain during the act. She puts her hands to her genitals frequently, but the mother is positive that it is for no vicious purpose.

About three years ago her general health began to improve, and has continued to do so until now, when we find her a well-nourished girl of average size. About four weeks ago a leucorrhœal discharge was noticed, which continued to increase for three weeks, at which time blood was mixed with it. Both discharges, however, has ceased. The mother thinks the child is somewhat backward intellectually. As you now observe she is unable to stand alone. When we ask her to move the lower extremities she makes many efforts before she succeeds, and then only slightly raises the thigh. When she endeavors to walk the limbs are moved altogether at the hip, while the thighs are strongly adducted, and as she brings one limb in front of the other it crosses in front of its fellow. When the other is brought forward it strikes on the opposite limb behind. The upper extremities also lack in the co-ordinating power, as you see the movements of her arms are slow, halting and uncertain. She has a peculiar unintelligent, silly look. We are unable to fix her attention on anything long at a time. She soon gets out of patience, cries, and the next moment bursts out into a laugh—laughs while the tears still trickle down her cheeks - showing in a very marked degree what is known as hysteria. Having bared her lower extremities, you will observe that her limbs are of good size, the thighs slightly flexed, and strongly adducted, and the legs are flexed on the thighs. The feet are in extreme extension, forming talipes equinus. Anterior to the mediotarsal joints the feet are turned inwards, forming the deformity to which we apply the name of varus.

When I come to examine the plantar surfaces I find the arch deepened and shortened, and the fascia very tense. So here there is a double deformity, an equinus and varus. When two deviations occur in the foot at the same time, the names are associated, prefixing that one which represents the greater deformity; so in this case, the equinus being the greater, the deformity is called talipes equino-varus.

The deformity is much more marked in the right foot, and you can notice this hard projection in front of the tibia, which is the scaphoid subluxated. If I ask the child to separate her thighs, she makes some effort but fails to get control of her muscles. By taking hold of the knees and making an effort to separate them for her, I find it takes considerable force, gradually applied, to make abduction to any extent, the muscles being rigid and unyielding. I find the muscles of both lower extremities in the same condition. Having flexed the leg upon the thigh at an angle of about 45°, so as to relax the extensors of the foot, I will see how near the foot can be restored into its normal position. As you see, by great force, gradually applied, I am enabled to bring the foot at right angles with the leg, or nearly so. By increasing the flexion of the leg, so as to relax the gastrocnemius and soleus as much as possible, I can carry the extension of the foot still a little farther. I have continued this now as far as possible. You will now ask the question, and justly so, if the extensors can be stretched thus far, why not farther—gradually, until they allow the foot to assume its shape, and the deformity is cured.

This question is easily decided by the following test. Having carried extension as far as possible, I have put the tendo-Achillis tightly upon the stretch. While holding it in that position, I take my thumb and make additional pressure on it, and, if under this additional tension there is no reflex. spasm, you can stretch it still further, and a cure may and can be effected without tenotomy; but, if, on the contrary, that additional pressure produces reflex spasm at the time, tenotomy must be performed, as you can never cure your case without it. You see the additional tension produces immediately a reflex spasm, and therefore we shall perform tenotomy. In bringing both tendo-Achilles under this test, also the plantar fasciæ, we find that they give the reflex spasms, and must be divided. When you obtain this test, you may know structural change has taken place, and no amount of tension, however applied, will ever restore the parts into their normal position.

The assistant will now proceed to give the child chloroform, but before doing so I will see that her clothing is not constricting her neck, chest, or waist. This is a very important point to look after befor the administration of an anæsthetic, and I advise you to never neglect it when you come to practice your profession.

I now show you the instrument I shall use to divide these tendons and faseiæ—my tenotome. The instruments you will find for sale in most of the establishments are a sharp-pointed straight knife. Never use one of them under any consideration. They are dangerous.

The next objection is that they have a straight cutting edge. This instrument you see has a convex cutting edge and a concave strong back. It is very narrow, not exceeding a line, and has a broad convex point. Here is an instrument that claims to be an exact counterpart of mine, but you see the blade is twice as wide. I have never met with an accident in dividing the muscles, tendons, and fasciæ, and I attribute no little to the instrument I use. If you use a sharp-pointed tenotome, if a vessel or nerve comes before it, you are certain to puncture it, but with this rounded point, which is not very sharp, such tissues will slip away before it.

The patient having become completely narcotized, I shall proceed with the operation.

Turning the patient over on her side, so as to turn the plantar surface upward, I have the fascia put tightly on the stretch by an assistant grasping the front part of the foot, and, making strong extension, I search for the inner border of the plantar fascia, and, having found it, I introduce the tenotome flatwise through the skin, and now I am able to feel the inner border of the fascia with the point of my knife. I gradually sink my instrument until I feel I am under the fascia. Then I pass the knife horizontally under it until I have reached the other side. I now turn the cutting edge of the instrument upward against the fascia, and my thumb is placed over it. I press the fascia on the knife, at the same time giving the latter a gentle to-an-fro motion until all the contractured tissues are divided. I turn the knife on the flat again and withdraw it, and as I do so I follow it with my thumb and squeeze out what blood there may be in the wound and guard against the ingress of air. On removing my thumb I apply a piece of adhesive plaster quickly, so as to hermetically seal the wound; over this I place some cotton wool, and secure both with a roller. The assistant now renders the tendo-Achillis tense by forcibly extending the foot, while I divide the latter just as I did the plantar fascia. After having divided, hermetically sealed, and applied the cotton and roller, as above described, the whole foot, excepting the toes, is enveloped in cotton wool, to guard against injurious compression, and a roller is applied over the foot and ankle for about five or six inches above the latter. An extra thickness of cotton should be applied to the sole of the foot.

I now bring the foot straight on into its normal position, which you see, the foot being at right angles with the leg, and as I cannot sit here and hold it until the result of the operation shall be repaired, I shall endeavor to accomplish the same end by the following dressing: Take a thin board, such as a shingle or the lid of a cigar box, and cut it so that it shall be about a quarter of an inch wider, and about three inches longer than the foot, modelling it after the shape of the latter. Then take a strip of adhesive plaster about two and a half inches wide, and of sufficient length to surround the foot board, and reach above the knee. Now take the board into your hand, the heel towards you, and wind the adhesive plaster around it lengthwise, commencing within an inch or two of the front of the board on the under surface (the adhesive side being next to the board). Bring it forward over toe end, back over the heel end, and then forward on the under side to the front, catching the end where you started. After having wrapped the board in this manner, your adhesive plaster should still be sufficiently long to reach half way up the thigh.

Take another strip of adhesive plaster from one to two feet long, owing to the size of the foot, and an inch or two wide, and fasten it about its middle to the heel of the board, in such a manner, that about half the width of the plaster will catch the under surface of the heel of the board. I do this in order to get a good hold so that it cannot slip off. Now apply a thick layer of cotton on the board that is to go next to the foot, and apply a roller pretty firmly, so as to prevent the cotton from slipping. This always should be done, so as to protect the bottom of the foot from pressure. In many of these cases the tissues about the feet are extremely insensible to irritants, and having a low vitality extensive sloughing might be the result from applying the board if not well cushioned.

Now apply the cushioned surface of the board to the sole of the foot, allowing the heel of the board to project an inch beyond that of the foot. While holding the foot at right angles with the leg, and the board firmly against the bottom of the foot, take one end of the plaster fastened to the heel of the board, bring it forward across the dorsum of foot to the opposite side of the foot from where you started, and pass it over the edge of the board to the under surface, where you fasten it. Then take the other end of the same plaster and follow the same movements, except that you carry it to the opposite side. After having completed this sandal-like fastening you take a roller and firmly secure the "foot-piece" to the foot. Should the foot have a tendency to turn in or out, an extra strip of adhesive plaster becomes necessary.

In this child, you see, the foot is inclined to turn inwards, and, therefore, I shall take this strip of plaster, which is about one and a half inches wide and a foot and a half long, start with one end on the outer side and front part of the foot, pass it across the dorsum toward the inner border under the foot and bring it to the outer edge and secure it with a few turns of a roller. The wide strip of adhesive plaster at the front of the board is now carried up the leg to the knee, or, where a good hold can be obtained, as in very little children, half way up the thigh. The guy is now carried up on the outer or inner side of the leg, as the case may be, and tension applied until the lateral deviation is corrected. The strip carried up the front is applied to the leg, while the foot is at right angles—both are secured to the leg by a roller. The extra lengths of the plasters are reversed, which causes the dressing to remain in place longer. One caution is necessary in applying this dressing, viz., *that you leave the toes exposed so you can always keep watch of the circulation.*

Having completed the dressing of this, the right foot, I shall proceed to the left one, where I shall divide the same tissues and apply a similar dressing.

You see both feet dressed, and perfectly straight. Were I now to put the child in the erect posture she could stand squarely on the soles of her feet. This is a simple, inexpensive dressing, obtainable anywhere, and for effectiveness is superior to any or all the dressings I know anything about.

Formerly surgeons did not treat their cases as we have this one to-day. I refer to the correction of the deformity *immediately* after the sections. It was the practice, and I am exceedingly sorry to say is to a great extent still, to allow the foot to remain in its deformed position for a number of days, until the external wound has cicatrized, and the division of the tendon repaired to a slight degree. As soon as the surgeon thought that glutinous material had been thrown out between the ends of the tendons and began to organize, he then, and not until then, could commence to correct the deformity. He commenced to apply traction, in order to stretch this new material that nature was throwing out between the ends of the tendon. This he was to continue until he had cured the deformity. I have followed this plan myself years ago, but it was tedious, caused the patient untold misery, and seldom succeeded. In the few cases where it did succeed in correcting a deformity, the stretched tendon was too feeble to give proper support.

Meeting with so much difficulty with this method of treatment, and the permanent good occomplished being so seldom satisfactory, I finally concluded to abandon it. I determined to restore the parts immediately after performing tenotomy, being certain the result could not be much worse if the divided ends never united than where the union was so feeble. But to my great surprise and satisfaction I discovered that, after ten or twelve days, the patient could move his foot by bringing into action the muscle of which the tendon had been severed—clearly showing that union was possible while the divided ends were separated. Since then I have abandoned the old for the new method, and after many years of a large field of experience, I pronounce the latter method as the proper one.

This is to me an extremely interesting case. I have shown you several cases during the last winter in my clinic, very similar to this, in male children due to congenital phimosis. In them, as in this little girl, there was loss of co-ordination, and paresis and rigidity of the muscular tissue of the lower extremities, the nervous system exhausted, and the mind much impaired, amounting in one case to an idiotic condition. In none of our cases, however, have we noticed the hysterical condition that we see in this little girl. What is the condition of this little girl due to? I believe it to be due to genital irritation, and I also believe that the source of irritation has been the presence of the oxyuris in the genital tract.

The future treatment of this patient will be to remove the source of irritation of the genital organs by destroying, if possible, the parasite which produces it, and to restore muscular power.

I shall leave on this dressing for eight or ten days, and then remove it, when I shall expect to see all the incisions firmly united. The feet will then be redressed for a week or two longer, until the tendons and fasciæ have firmly united. I shall then maintain the feet in proper position by the aid of properly fitting shoes,and elastic force if necessary, while I restore the muscles to a healthy condition by the use of massage, properly directed gymnastic exercise, electricity, etc, etc.

Cases II. and III.—I have next to show you a pair of male twins. The mother brought them to my office some two weeks ago to consult me in regard to a hernia, which had been developed in each since their birth. I found an inguinal hernia in each on the same side. While examining the hernia of the first child I discovered that the penis was unusually large, and in a state of semi-erection. On questioning the mother she stated that the penis was in erection a great deal of the time; that the children cried a great deal; that when they urinated they strained very hard, and screamed as though they were suffering extremely. Both were in the same condition. On closer examination the opening in the prepuce was found extremely small, and it was with great difficulty that the mouth of the urethra could be seen. The latter was very red and angry looking, and on being touched a spasm of the lower extremities followed. Behind the corona glandis there was noticed a large, hard swelling.

There is not the least doubt in my mind, gentlemen but that the hernia is a result of the phimosis. The very small opening in the prepuce which prevented a free discharge of urine, and the extremely irritable condition of the mouth of the urethra, causing excruciating suffering every time urine passed over it, were the cause of the crying and straining, which brought on the hernia.

I consider it for these children extremely fortunate that this genital irritation has been discovered so early—before it has had time to seriously enervate the nervous system with all its evil consequences. In order to prevent any further ill effects on the system, it will be necessary to free the glans penis in each one by an operation.

I will now perform the operation of circumcision. I catch hold of the end of the prepuce with my fingers and draw it forward. I now place a pair of Henry's forceps in front of the glans, grasping the prepuce. When I have drawn through the amount of prepuce I wish to excise, I firmly close the blades of the forceps and lock them. Having done this I take a sharp bistoury and cut off the prepuce in front of the blades of the forceps. I now unlock the forceps and free the prepuce As you can all see, I have cut off the outside skin only, the mucous membrane remaning. I have not enlarged the opening in the prepuce in the least, and the glans is as much imprisoned as before.

I have seen several cases where the surgeon, when he had gone thus far, thought he had completed the operation. Only a few days ago a boy, eight years old, was brought to me from Florida, where this was the case. After many months cicatrization took place, and left the child worse than he was before. The cicatricial tissue had contracted, and was hard and unyielding. To avoid any such bad consequences, you now see me pass this small grooved director into the preputial opening in front up to the glans. You see I do this with considerable difficulty, owing to the agglutination of the prepuce and glans. I am unable to carry the director entirely up to the corona, consequently I shall divide the mucous membrane as far as the director has been passed. Having done this I now separate the mucous membrane from the glans, by tearing it loose, with my thumb and fingers.

Having entirely separated the glans and prepuce, you see, the corona is very red, and behind it I find hardened, gritty, smegma. Having completed the operation thus far, I want next to call your attention to an extremely important part of the procedure, viz., the frænum. This you will usually find very short, with pockets on either side.

Never consider your operation completed until you have broken down this shortened band of mucous membrane. Should you neglect it your operation will, in all probability, remain without any benefit to the patient. Having broken down the band of mucous membrane, the frænum, see that neither it nor the outside skin in any way constricts the penis—*if there are any constricting bands they must be divided.* The operation being completed I shall dress it as follows: In very small children I seldom use sutures, but bring the cut edges of the skin and mucous membrane accurately together, behind the glans, and then take a soft piece of old linen cloth, or lint, and wrap around the penis over the coaptated cut edges. I allow the meshes of the cloth to become saturated with blood, and if hemorrhage does not cease I appply over this some styptic cotton. Then I form a circular pad, with a hole through the centre, into which the penis is placed and allowed to remain, excepting when the dressings are renewed, until cicatrization is complete. A pledget of lint dipped into water is placed over the glans, and the whole retained in place by a diaper. This dressing will remain on for five or six days, when I shall expect union to have taken place.

There is one thing I want to caution you against, and that is, if cicatrization should be delayed, to guard against subsequent adhesion of the prepuce to the glans, or the formation of cicatricial bands. Many a surgeon has met with failures by not observing this precaution.

*Note.* May 1, 1880.—The first case, that of the little girl, has improved beyond all expectation. The dressings of both feet were removed on the eighth day, and very fortunate it was. Although the feet were protected from pressure by thick layers of cotton-wool, if the dressing had been allowed to remain on a few days longer extensive sloughing on the instep would have been certain. As it was, only superficial destruction of the skin over a small area took place. The same dressing was reapplied, and changed once in two or three days for three weeks. Properly-fitting shoes were then applied (ordinary shoes made to fit the child), which retained the feet in proper position without any elastic force. Since then she has had electricity applied every other day, and the muscles of the thighs and legs have been manipulated daily, the peronei receiving especial attention. The general health has been improved by proper dieting and plenty of outdoor exercise. The patient has received daily training in the use of her muscles, and especial effort has been exerted to "hitch her brain to her muscles"—to improve her will-power, and inspire confidence in her own capabilities.

She is now able to abduct her thighs to their fullest capacity—her limbs are perfectly straight. They have increased in size and strength. She is now able to walk across the room. When the feet are bared, they can be held perfectly straight; she can extend and flex them very nearly perfectly. When they are carried to extreme extension the feet turn slightly inwards, showing a deficiency of power in the peronei muscles. F. G.

